# The stress signalling pathway nuclear factor E2-related factor 2 is activated in the liver of sows during lactation

**DOI:** 10.1186/1751-0147-54-59

**Published:** 2012-10-05

**Authors:** Susann Rosenbaum, Robert Ringseis, Sonja Hillen, Sabrina Becker, Georg Erhardt, Gerald Reiner, Klaus Eder

**Affiliations:** 1 Institute of Animal Nutrition and Nutritional Physiology, Justus-Liebig-University, Heinrich-Buff-Ring 26-32, Giessen, 35392, Germany; 2 Department of Veterinary Clinical Sciences, Swine Diseases, Justus-Liebig-University, Frankfurter Straße 112, Giessen, 35392, Germany; 3 Institute for Animal Breeding and Genetics, Justus-Liebig-University, Ludwigstraße 21b, Giessen, 35390, Germany

**Keywords:** Sow, Liver, Lactation, Inflammation, Nrf2 pathway, Acute phase proteins

## Abstract

**Background:**

It has recently been shown that the lactation-induced inflammatory state in the liver of dairy cows is accompanied by activation of the nuclear factor E2-related factor 2 (Nrf2) pathway, which regulates the expression of antioxidant and cytoprotective genes and thereby protects tissues from inflammatory mediators and reactive oxygen species (ROS). The present study aimed to study whether the Nrf2 pathway is activated also in the liver of lactating sows.

**Findings:**

Transcript levels of known Nrf2 target genes, *UGT1A1* (encoding glucuronosyltransferase 1 family, polypeptide A1), *HO-1* (encoding heme oxygenase 1), *NQO1* (encoding NAD(P)H dehydrogenase, quinone 1), *GPX1* (encoding glutathione peroxidase), *PRDX6* (encoding peroxiredoxin 6), *TXNRD1* (encoding thioredoxin reductase 1), and *SOD* (encoding superoxide dismutase), in the liver are significantly elevated (between 1.7 and 3.1 fold) in lactating sows compared to non-lactating sows. The inflammatory state in the liver was evidenced by the finding that transcript levels of genes encoding acute phase proteins, namely haptoglobin (HP), fibrinogen γ (FGG), complement factor B (CFB), C-reactive protein (CRP) and lipopolysaccharide-binding protein (LBP), were significantly higher (2 to 8.7 fold) in lactating compared to non-lactating sows.

**Conclusions:**

The results of the present study indicate that the Nrf2 pathway in the liver of sows is activated during lactation. The activation of Nrf2 pathway during lactation in sows might be interpreted as a physiologic means to counteract the inflammatory process and to protect the liver against damage induced by inflammatory signals and ROS.

## Findings

Typical characteristics for the lactation phase are dramatic increases in the energy and nutrient requirement of the organism, which are usually met by an elevation of food intake and a mobilisation of body’s energy stores [1,2]. In dairy cows, lactation was also shown to induce immune and inflammatory responses in the liver [3,4]. In contrast to extensive research in rodents and cows on the mechanisms underlying the lactation-induced metabolic and immunologic adaptations [3-6], only limited information is available in sows in this regard [7,8]. In an attempt to improve the knowledge about metabolic and immunologic adaptations during lactation in sows we have recently analyzed the changes in the hepatic transcriptome of sows during lactation on a genome-wide level [9]. Besides the expected induction of energy-generating pathways in the liver during lactation, we found that lactation causes an induction of an inflammatory state as evidenced from the marked induction of several acute phase proteins in the liver of lactating sows [9]. Acute phase proteins are secreted from hepatocytes in response to inflammatory stimuli and are therefore established markers of inflammation in both human and veterinary clinical pathologies, which indicates that lactation induces an inflammatory state in the liver of sows, like in dairy cows [3,4]. Interestingly, we have recently reported that the inflammatory state in the liver of dairy cows during lactation is accompanied by activation of the nuclear factor E2-related factor 2 (Nrf2) stress signalling pathway (unpublished observations). Nrf2 is a redox-sensitive transcription factor which upon activation by inflammatory stimuli but also reactive oxygen species (ROS), xenobiotics or electrophiles [10] induces the transcription of a large set of genes encoding various antioxidative proteins such as glutathione peroxidase 1 (encoded by *GPX1*), superoxide dismutase (encoded by *SOD*) and cytoprotective proteins [heme oxygenase 1 (encoded by *HO-1*), NAD(P)H dehydrogenase, quinone 1 (encoded by *NQO1*), peroxiredoxin 6 (encoded by *PRDX6*), thioredoxin reductase 1 (encoded by *TXNRD1*), glucuronosyltransferase 1 family, polypeptide A1 (encoded by *UGT1A1*)] and thereby protects tissues from inflammatory damage and neutralizes ROS produced under pro-inflammatory conditions [11]. In light of the observation that the lactation-induced inflammatory state in the liver of dairy cows is accompanied by activation of the Nrf2 pathway and in order to further improve knowledge about the mechanisms underlying the lactation-induced adaptations during lactation in sows, the present study aimed to investigate whether the Nrf2 pathway is activated in the liver of lactating sows.

The animal study was carried out in accordance with established guidelines for the care and handling of laboratory animals and was approved by the local Animal Welfare Authorities (Regierungspräsidium Giessen; permission no: GI 19/3-No. 29/2010). As described recently in more detail [9], the experiment was performed with twenty second parity sows (Large White & German Landrace), which were artificially inseminated with semen from boars of the own breed, and kept in single crates until day 21 of pregnancy. From day 21 to 110 of pregnancy, the sows were kept in groups in pens that had fully slatted floors, nipple drinkers and feeding stations. On day 110 of pregnancy, they were moved to the farrowing accommodation where they were housed in single farrowing pens. Throughout pregnancy, sows of both groups were fed a commercial diet for gestating sows *ad libitum*. After farrowing, the sows were randomly assigned into two groups of 10 animals each. In the first group of sows, all piglets were removed from the sow (“non-lactating group”) 24 h after parturition. This group served as the non-lactating control. In the second group, litters were standardised to 12 piglets per sow (“lactating group”). Throughout lactation until the end of the experiment the sows received a diet for lactating sows. Until day 6 after farrowing, the amount of feed given to the lactating sows was successively increased, and from day 7 after farrowing and thereafter the sows were fed individual amounts of feed depending on their body weights. In the non-lactating group, each sow received an amount of food sufficient to cover the individual energy and nutrient requirement for maintenance. On day 20 after farrowing, liver tissue was taken by biopsy and blood was collected from *Vena jugularis*. A full description of the housing condition, diet composition, feeding regime and sample collection including biopsy sample procedure can be found in our recent publication [9]. RNA isolation from frozen liver biopsies and quantitative real-time PCR (qPCR) analysis were performed as described recently in detail by Keller *et al.*[12]. Characteristics of primers and primer performance data used for qPCR analysis are shown in Table 1. Plasma levels of TBARS were measured in plasma using a modified version of the TBARS assay [13]. Statistical analysis was performed by one way analysis of variance. Fisher’s multiple range test was used to generate significant F-values of differences with *P* < 0.05.

**Table 1:** Characteristics of primers and primer performance data used for qPCR

**Gene symbol**	**Forward primer (from 5’ to 3’)**	**Product size (bp)**	**NCBI GenBank**	**Slope**	**R2**^**#**^	Efficiency*	**M**
	**Reverse primer (from 5’ to 3’)**						
*Reference genes*							
RSP9	GTCGCAAGACTTATGTGACC	325	XM_003356050	-0.28	0.999	1.91	0.053
	AGCTTAAAGACCTGGGTCT						
ATP5G1	CAGTCACCTTGAGCCGGGCA	94	NM_001025218	-0.30	0.998	1.99	0.054
	TAGCGCCCCGGTGGTTTGC						
GSR	AGCGCGATGCCTACGTGAGC	175	AY368271	-0.29	0.997	1.94	0.055
	GGTACGCCGCCTGTGGCAAT						
ACTB	GACATCCGCAAGGACCTCTA	205	XM_003124280	-0.32	0.992	2.1	0.064
	ACATCTGCTGGAAGGTGGAC						
SHAS2	GAAAAGGCTAACCTACCCTG	218	NM_214053	-0.21	0.996	1.65	0.076
	TGTTGGACAAGACCAGTTGG						
*Target genes*							
HP	ACAGATGACAGCTGCCCAAA	188	NM_214000	-0.30	0.997	1.99	
	CCGCACACTGCTTCACATTC						
FGG	GACATCTGTCTCCTACTGGA	375	NM_001244524	-0.29	0.999	1.95	
	CATGACACTTGTTCATCCAC						
CFB	CTCAACGCAAAGACCGCAAA	106	NM_001101824	-0.29	0.998	1.96	
	AAATGGGCCTGATGGTCTGG						
CRP	CCTTTGTCTTCCCCAAAGAG	563	NM_213844	-0.28	0.999	1.91	
	CACCTCGCCACTCATTTCAT						
LBP	ACCGCTCCCCAGTTGGCTTC	406	NM_001128435	-0.29	0.999	1.96	
	AGCGCGGCGGACACATTAGT						
NQO1	CCAGCAGCCCGGCCAATCTG	160	NM_001159613	-0.28	0.997	1.89	
	AGGTCCGACACGGCGACCTC						
TXNRD1	CTTTACCTTATTGCCCGGGT	162	NM_214154	-0.30	0.999	1.98	
	GTTCACCGATTTTGTTGGCC						
UGT1A1	GATCCTTTCCTGCAACGCAT	313	XM_003483776	-0.28	0.996	1.91	
	GGAAGGTCATGTGATCTGAG						
HO-1	AGCTGTTTCTGAGCCTCCAA	130	NM_001004027	-0.30	0.998	1.98	
	CAAGACGGAAACACGAGACA						
PRDX6	GGCCGCATCCGTTTCCACGA	280	NM_214408	-0.29	0.998	1.95	
	ACTGGATGGCAAGGTCCCGACT						
SOD	TCCATGTCCATCAGTTTGGA	250	NM_001190422	-0.27	0.998	1.88	
	CTGCCCAAGTCATCTGGTTT						
GPX1	GGCACAACGGTGCGGGACTA AGGCGAAGAGCGGGTGAGCA	235	NM_214201	-0.29	0.998	1.96	

^#^Coefficient of determination of the standard curve. *The efficiency is determined by [10^-slope^].

Abbreviations: *RSP9* 40S ribosomal protein S9-like, *ATP5G1* ATP synthase, H+ transporting, mitochondrial Fo complex, subunit C1 (subunit 9), *GSR* glutathione reductase, *ACTB* actin, beta, *SHAS2* hyaluronan synthase 2, *HP* haptoglobin, *FGG* fibrinogen γ, *CFB* complement factor B, *CRP* C-reactive protein, *LBP* lipopolysaccharide-binding protein, *NQO1* NAD(P)H dehydrogenase, quinone 1, *TXNRD1* thioredoxin reductase 1, *UGT1A1* glucuronosyltransferase 1 family, and polypeptide *A1; HO-1* heme oxygenase 1, *PRDX6* peroxiredoxin 6, *SOD* superoxide dismutase, *GPX1* glutathione peroxidase 1.

As expected, evaluation of body weight development of sows throughout lactation revealed that sows of both groups lost body weight but that body weight loss was greater in lactating than in non-lactating sows despite the fact that lactating sows had a markedly greater feed intake [9]. Estimation of the energy balance of the sows revealed that lactating sows are in a strong negative energy balance during lactation indicating that the increased feed intake was not sufficient to fully compensate the body weight loss. In order to evaluate activation of Nrf2 pathway in the liver of sows we determined transcript levels of several known Nrf2 target genes in the liver samples. The results of the present study clearly show that the transcript levels of known Nrf2 target genes, *UGT1A1*, *HO-1*, *NQO1*, *GPX1*, *PRDX6*, *TXNRD1*, and *SOD*, in the liver are markedly elevated (between 1.7 and 3.1 fold) in lactating sows compared to non-lactating sows (*P* < 0.05, Table 2). Given that the above mentioned genes contain functional antioxidant response elements (ARE), which are the DNA binding motifs for Nrf2, in their regulatory regions [14], the up-regulation of these genes during lactation indicates, at least indirectly, that the Nrf2 pathway is indeed activated in the liver of lactating sows. This is in line with recent observations in dairy cows in which the Nrf2 pathway was shown to be strongly activated in liver (unpublished observations) and spleen [15] in early lactation compared to late pregnancy. Due to the limited amount of liver tissue obtained from the biopsy sampling procedure additional assays, such as gel-shift assays providing direct evidence for activation of Nrf2 through determining of binding of Nrf2 to the ARE motif of Nrf2 target genes, could not be conducted. Due to the same reason protein levels of the Nrf2 target genes were not determined. However, several studies clearly showed that increased mRNA levels of Nrf2 target genes positively correlate with elevated levels of the encoded proteins [16,17], suggesting that elevated Nrf2 target gene levels are suitable indicators of Nrf2 activation.

**Table 2:** Relative transcript levels of Nrf2 target genes in the liver of lactating and non-lactating sows on day 20 of lactation

	**Non-lactating (n = 10)**	**Lactating (n = 10)**	***P*value**
UGT1A1	1 ± 0.52	1.79 ± 0.59*	0.013
HO-1	1 ± 0.48	1.71 ± 0.42*	0.007
NQO1	1 ± 0.49	2.91 ± 1.54*	0.007
GPX1	1 ± 0.56	3.11 ± 1.19*	0.001
PRDX6	1 ± 0.56	2.12 ± 0.78*	0.008
TXNRD1	1 ± 0.28	2.99 ± 2.45*	0.029
SOD	1 ± 0.65	2.28 ± 1.01*	0.007
MT1A	1 ± 0.37	1.64 ± 1.26	0.190

Values represent mean ± SD for n = 10 sows per group. *Significantly different from non-lactating group (*P* < 0.05).

With regard to the mechanism underlying the activation of Nrf2 during lactation, it may be speculated that ROS, which are known activators of Nrf2 [10] and generated during an inflammatory process and an elevated energy production through the respiratory chain, are the stimuli triggering the activation of Nrf2 in the liver during lactation. Transcript levels of genes encoding acute phase proteins, such as haptoglobin (encoded by *HP*), fibrinogen γ (encoded by *FGG*), complement factor B (encoded by *CFB*), C-reactive protein (encoded by *CRP*) and lipopolysaccharide-binding protein (encoded by *LBP*), were clearly higher (2 to 8.7 fold) in lactating compared to non-lactating sows (*P* < 0.05, Table 3) providing at least indirect evidence for an inflammatory state occurring in the liver of lactating sows. In addition, the recently observed induction of energy-generating pathways (fatty acid catabolism, tricarboxylic acid cycle, respiratory chain) in livers of lactating sows [9] is supportive for an elevated energy production during lactation. The unaltered plasma levels of TBARS, which were used as markers of oxidative stress, between lactating and non-lactating sows in the present study (7.03 ± 1.54 vs. 7.02 ± 2.25 nmol/μmol triacylglycerols, n = 10/group, *P* > 0.05) suggests at first glance that ROS production was not elevated in sows during lactation. However, the lack of an increase in TBARS levels may be simply explained by the up-regulation of antioxidant genes which likely improved the capacity of the liver to cope with the increased levels of ROS and to prevent oxidative stress.

**Table 3:** Relative transcript levels of genes encoding acute phase proteins in the liver of lactating and non-lactating sows on day 20 of lactation

	**Non-lactating (n = 10)**	**Lactating (n = 10)**	***P*value**
HP	1 ± 0.89	2.58 ± 1.08*	0.005
FGG	1 ± 0.55	2.90 ± 0.66*	0.001
CFB	1 ± 0.52	2.01 ± 0.36*	0.001
CRP	1 ± 0.68	8.68 ± 4.20*	0.001
LBP	1 ± 0.71	5.82 ± 1.94*	0.001

Values represent mean ± SD for n = 10 sows per group. *Significantly different from non-lactating group (*P* < 0.05).

**Figure 1:**
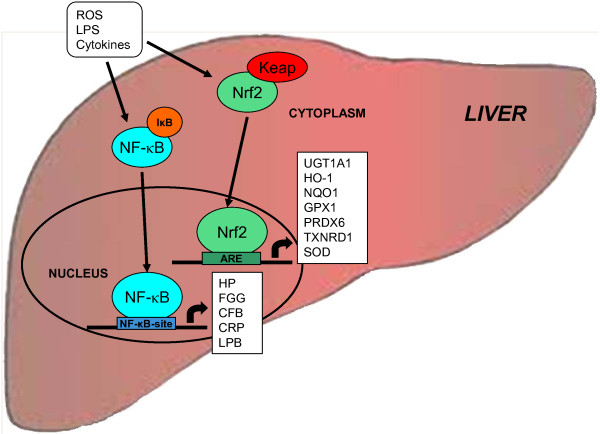
**Activation of stress signalling pathways in the liver of sows during lactation.** Activation of Nrf2 and NF-κB during lactation is mediated by various stimuli including reactive oxygen species (ROS), bacterial lipopolysaccharide (LPS) and cytokines, which are known activators of both, Nrf2 and NF-κB. Activation of Nrf2 leads to up-regulation of classical Nrf2 target genes such as glutathione peroxidase 1 (encoded by *GPX1*), superoxide dismutase (encoded by *SOD*) and cytoprotective proteins [heme oxygenase 1 (encoded by *HO-1*), NAD(P)H dehydrogenase, quinone 1 (encoded by *NQO1*), peroxiredoxin 6 (encoded by *PRDX6*), thioredoxin reductase 1 (encoded by *TXNRD1*), glucuronosyltransferase 1 family, and polypeptide A1 (encoded by *UGT1A1*)]. Acivation of NF-κB results in the induction of acute phase proteins such as haptoglobin (encoded by *HP*), fibrinogen γ (encoded by *FGG*), complement factor B (encoded by *CFB*), C-reactive protein (encoded by *CRP*) and lipopolysaccharide-binding protein (encoded by *LBP*). Activation of stress signalling pathways during lactation in sows might be interpreted as a physiologic means to counteract the inflammatory process and to protect the liver against deleterious effects of inflammatory signals and ROS, which are released at elevated levels as a consequence of the metabolic and immunologic adaptations occurring during the transition from pregnancy to lactation.

In conclusion, the results of the present study indicate that the Nrf2 pathway in the liver of sows is activated during lactation. The activation of Nrf2 pathway during lactation in sows might be interpreted as a physiologic means to counteract the inflammatory process and to protect the liver against damage induced by inflammatory signals and ROS, which are released at elevated levels as a consequence of the metabolic and immunologic adaptations occurring during the transition from pregnancy to lactation (Figure 1). Since Nrf2 is also expressed in the mammary gland, which is particularly susceptible for the induction of an inflammatory state caused by bacterial invasion via the mammary gland [18], it is likely that activation of this pathway during lactation might provide protection against bacterial derived inflammatory stimuli also in non-hepatic tissues.

## Competing interests

The authors declare that they have no competing interests.

## Authors' contributions

SR conducted the animal experiment, performed the PCR analyses and the statistical analyses and wrote the manuscript. RR supervised PCR analyses and helped to draft the manuscript. SH and GR established and supervised the liver biopsy sampling procedure. SB conducted liver biopsy sampling. GE was responsible for animal keeping. KE conceived of the study, participated in its design and coordination and helped to draft the manuscript. All authors read and approved the final manuscript.
